# Accuracy of the lower third molar radiographic imaging to estimate age among Ugandan young people

**DOI:** 10.1186/s13104-019-4686-1

**Published:** 2019-10-11

**Authors:** Catherine Lutalo Mwesigwa, Annet M. Kutesa, Ian G. Munabi, Catherine A. Kabenge, William Buwembo

**Affiliations:** 1 Department of Dentistry, School of Health Sciences, College of Health Sciences, Makerere University, P.O.Box 7072, Kampala, Uganda; 2 Department of Anatomy, School of Biomedical Sciences, College of Health Sciences, Makerere University, Kampala, Uganda; 3 Department of Oral & Maxillofacial, Mulago National Referral Hospital, Kampala, Uganda

**Keywords:** Dental age estimation, Third molar mineralisation, Forensic odontology, Demirjian, Specificity, Sensitivity, Uganda

## Abstract

**Objective:**

Dental development is a useful method for age estimation. Although third molar eruption is commonly used to estimate age in Uganda, it is reported to be unreliable because of external influences. The more reliable radiographic techniques have inter-ethnic differences but data from sub-Saharan Africa are limited regarding estimating age in young adults. This study, therefore, aimed at determining the accuracy of Demirjian’s classification of the lower third molar, a common dental age estimation method, in estimating key ages in a Ugandan population using Ugandan references. Dental records of 1021 Ugandans aged 10–22 years were assigned to two groups; reference and test. The reference data was retrieved from a database of a previous bigger research project.

**Results:**

The overall sample population comprised of 514/1021 (50.3%) males. The mean age was 15.8 (3.6) years. No significant sex differences in dental age were established in the reference sample (520 records). Accuracy values (area under the curve) at the 12-, 14-, 16- and 18-year cut-offs were between 0.83 and 0.90 using the test sample (501 records). The results suggest that Demirjian’s classification of the lower third molars is a useful method for age estimation in the young urban Ugandan population in the 10–22-year age-group.

## Introduction

Assessment of dental development is a useful tool for age estimation among adolescents and young adults [1]. This is important when an individual’s age cannot be verified for decision making [1, 2]. Unpublished observations by Kutesa MA indicate that to estimate ages of young Ugandans, the third molar is commonly visually inspected—a cheap, fast and non-invasive method. However, studies from other parts of the world have reported this method as unreliable because the timing of tooth eruption is generally affected by multiple factors like: environmental, nutritional status, local and systemic diseases [3–5].

In contrast, some research findings have revealed that radiographic techniques used for dental age estimation might not be affected by these factors including space availability and impaction [4, 6–8]. For visualisation, the dental panoramic radiograph (PAN), showing all teeth including non-erupted ones, is commonly used.

One of the commonest radiographic techniques, Demirjian’s method, was originally developed in a French–Canadian sample by scoring the seven left lower permanent [8]. It was later adapted to estimate age of older individuals using the third molar [9]. The method has eight ordinal stages of dental development. Age at a specific stage is derived by calculating the average age of individuals who show the stage in question [9].

Studies on age estimation using radiographic techniques have revealed population differences [9–11], and hence the need for population-specific references [12, 13]. However, data from sub-Saharan Africa are limited regarding the optimal dental age reference values for young adults, [10, 14–16] among whom third molars remain the only teeth useful for dental age estimation [17].

This study therefore, aimed to derive Ugandan dental age references using Demirjian’s classification based on the lower third molars, and to determine the accuracy of this method as a tool for discriminating the key ages of 12, 14, 16 and 18 years, in a Ugandan population [18–21].

## Main text

### Methods

Dental records of 1021 Ugandans aged 10–22 years were used for this study and included digital PANs from two types of urban/peri-urban populations in Kampala. One from the general population previously recruited for another study among individuals aged 5–25 years, with proof of their birth documentation. This population provided the “reference sample”. The second type of population was from patients attending three private urban clinics which were purposively selected on the basis of having patient records with digital PANs. The second population was assigned to the “test sample”. The inclusion criteria were Ugandans by ethnicity (self-report). A total of 1030 records were retrieved as determined from two separate sample size calculations. Records were picked randomly from each age group ensuring a minimum sample of 16 records per age group per sex. Groups with bigger numbers got more than 16 records sampled until the required sample size was attained.

The two intermediate sample size estimates were derived using published criteria for reference material [3, 17] and a nomogram for the test sample [22]. A nomogram is a graphic representation showing the relationships among several elements;- sample size, prevalence of a condition, confidence intervals, and specificity/sensitivity. If three of the elements are known, the fourth element is estimated. A nomogram has been used in sample size determination for diagnostic tests research because it is simple and can also be used retrospectively [22]. Each of the intermediate estimates was further increased by a factor of 0.2 to cater for the design effect from clustering within an individual [23], and 5% for missing teeth. The final estimates were 525 and 505 for the reference and test samples, respectively.

All the 1030 digital images were de-identified and saved in the JPEG format using a unique identifier number (UID) for future blinding of other data procedures. The other information on sex and chronological age (the difference in years between the date of birth and date of radiography) was saved as a separate excel file under each UID. The digital images were examined on a computer screen in a darkened room by two observers: a dental radiologist (CK) and a dentist (CLM), with 15 and 9 years’ experience, respectively. The maturation of the lower third molars was scored using the criteria according to Demirjian’s classification A to H. [8, 9]. The distal root was scored for stages G and H. For inter-observer and intra-observer agreement, 25% of randomly selected PANs were scored by both observers and another 25% were scored again after 2 months by observer CLM, respectively.

The scores were entered against the relevant UID. Records with unclear images and both third molars missing were dropped. The data on age, sex and Demirjian scores were merged, entered twice and validated using Epidata version 3.1. Finally, two separate data sets; the “reference data” and “test data” were obtained, details of which can be found in Additional files 1 and 2, respectively.

The age was computed in years without decimal points. Each third molar was analysed as a separate entity. Stata software version 13.0 (Stata, College Station, Texas, USA) was used for analysis. Data from the reference sample was used to determine dental age based on each third molar per sex by calculating the median age for each Demirjian stage. Mann–Whitney U test was applied to verify if there were sex differences in the dental age (*P* value of 0.05 for statistical significance). Thereafter, a combined dental age was put in a table as the reference value. The test sample was used for the subsequent analysis. The chronologic age was used as the “gold standard” while the dental age was the “test”. Sensitivity, specificity, Receiver Operator Curve (ROC) analysis, predictive values and likelihood ratios (LRs) at the different cut-off values of 12, 14, 16 and 18 years were obtained as described in a previous study [24]. For each age cut-off, a value of “1” (true) was assigned if one was that age or older, or “0” (false) if younger than the cut-off. The ROC analysis output was the Area under the Curve (AUC), which was used to interprete accuracy. The AUC ranges from 0 to 1, where “1” represents perfect discrimination such that as the AUC tends to 1, this suggests increased test accuracy. Swets further described AUC values between 0.9 to 0.7 as useful for some purposes [25].

The research protocol was reviewed and approved by the research ethics committees of the Makerere University School of Biomedical Sciences and Uganda National Council of Science and Technology.

### Results

The intra-observer and inter-observer Kappa values were 0.91 and 0.87, respectively, both indicating very good agreement. The 1021 sample population had an almost equal number of both sexes (514/1021 (50.3%) males) with overall mean and median ages of 15.8 (standard deviation (SD) = 3.6) and 16 (inter-quartile range = 6) years, respectively. Table 1 provides a summary of the other descriptive statistics of the two participant population groups. In the reference group almost equal numbers of males and females were included, while for the test group, the males were slightly more than the females.

**Table 1:** Characteristics of 1021 young Ugandans whose dental records were included in the study

	Reference group		Test group	
	Number of participants (%)	Mean years (SD)	Number of participants (%)	Mean years (SD)
Total	520 (100)	15.9 (3.8)	501 (100)	15.7 (3.5)
Females	262 (50.4)	15.9 (3.7)	245 (48.9)	15.7 (3.5)
Males	258 (49.6)	15.9 (3.9)	256 (51.1)	15.7 (3.4)
Age-group (years)				
10–12	128 (24.6)		115 (23.0)	
13–15	124 (23.8)		120 (24.0)	
16–18	115 (22.1)		144 (28.7)	
19–22	153 (29.4)		122 (24.3)	

SD is the standard deviation. A total of 1030 dental records were retrieved, six were excluded for having unclear lower third molars and three had both lower third molars with very short roots. The overall mean age was 15.8 (3.6) years

As shown in Table 2, reference values combined for the sexes were generated for the eight stages of Demirjian stages in the Reference sample. This was done only after finding that there were no significant sex differences. The reference values were similar for the right and left except at stages D and G, where dental age was lower on the right than the left by a year and half-a-year, respectively.

**Table 2:** Reference values for dental age estimation among 520 young Ugandans using Demirjian’s classification

Demirjian stage	Tooth 48 (n = 510)				Tooth 38 (n = 501)			
	No.	Median	LQ, UQ	Mean (SD)	No.	Median	LQ, UQ	Mean (SD)
A	2	10	10, 10	10 (0.0)	1	10	–	10 (0.0)
B	16	10	10, 11.5	10.9 (1.4)	13	10	10, 11.5	10.8 (1.2)
C	83	11	11, 12	11.7 (1.5)	85	11	11, 12	11.7 (1.5)
D	61	12	11, 14	12.6 (1.6)	62	13	11, 14	12.6 (1.5)
E	68	14	13, 15	13.9 (1.5)	62	14	13, 15	13.9 (1.6)
F	59	15	14, 16	15.4 (1.6)	63	15	14, 17	15.4 (1.7)
G	96	18.5	17, 20	18.4 (1.6)	93	19	17, 20	18.5 (1.6)
H	125	21	19, 22	20.1 (1.8)	122	21	19, 22	20.3 (1.7)

LQ and UQ represent lower quartile and upper quartile respectively. A–H are the eight stages of Demirjian’s classification. The difference between the sample number, 520 and number of teeth on each side (Right—510 and left—501) is accounted for by missing teeth or those that had not yet commenced mineralisation

Table 3 provides a summary of the sensitivity, specificity, predictive values, LRs, and AUC (and their confidence intervals) at the cut-offs of 12, 14, 16, and 18 years for both lower third molars. The AUC values at all cut-offs represent the accuracy of Demirjian’s classification to identify correctly an individual as the given cut-off age. All AUC values were ranging from 0.83 to 0.90. The highest AUC and sensitivity were at the 14-year cut-off while the lowest AUC and sensitivity were at the 16-year cut-off. The least specificity was at the 12-year cut-off. All positive LRs were greater than one.

**Table 3:** Accuracy of Demirjian’s classification of the lower third molar in estimating age of young Ugandans

Age cut-off (years)	Tooth	No.	Sensitivity (%)	Specificity (%)	AUC (95% CI)	PPV	NPV	+LR	−LR
12	38	496	92.0	80.3	0.86 (0.81 to 0.91)	0.97	0.63	4.7	0.1
	48	494	92.9	80.3	0.87 (0.82 to 0.91)	0.97	0.66	4.7	0.1
14	38	496	97.4	83.2	0.90 (0.87 to 0.93)	0.93	0.93	5.8	0.0
	48	494	97.4	82.9	0.90 (0.87 to 0.93)	0.93	0.93	5.7	0.0
16	38	496	68.8	97.4	0.83 (0.80 to 0.86)	0.97	0.73	26.7	0.3
	48	494	68.7	96.9	0.83 (0.80 to 0.86)	0.96	0.73	22.5	0.3
18	38	496	88.0	87.8	0.88 (0.85 to 0.91)	0.79	0.94	7.2	0.1
	48	494	85.9	86.7	0.86 (0.83 to 0.90)	0.77	0.92	6.5	0.2

No. is number of observations. The difference between the test sample number, 501 and number of teeth on each side (Right—494 and left—496) is accounted for by missing teeth or those that had not yet commenced mineralisation. PPV, NPV, +LRF and −LR are positive predictive value, negative predictive value, positive likelihood ratio and negative likelihood ratio, respectively

### Discussion

We set out to determine the accuracy of Demirjian’s classification of the lower third molar in discriminating between individuals of specific legal ages within the 10–22-year age-group in a Ugandan urban population. We found that the AUC at all cut-off ages based on the two-third molars were useful for some purposes, using Swets’ AUC scoring system [25].

The highest sensitivity observed at the 14-year-cut-off shows that Demirjian’s classification in this study population could most correctly identify individuals that have reached the age of 14 years. On the other hand, the highest specificity at the 16-year cut-off suggests that the third molars can be used to correctly classify individuals younger than 16 years. However, given that the same age group had both the lowest AUC and sensitivity shows that determining whether an individual has reached 16 years using this method alone should be done with caution.

The least accuracy could be due to the fact that between the stages F and G, which are representative of the 16-year-cut-off, there is a span of 3–3.5 years compared to the other cut-offs with 1–2 years representing the sequential stages. This ambiguity of the root stages has been alluded to as stemming from Demirjian’s method having only four root stages (E–H) representing a wide range of age distribution (14–20 years) [26]. Although having relatively fewer root stages makes Demirjian’s technique easy to use and reproducible, this may affect the accuracy of age estimation [27]. For this reason, Demirjian’s method was modified to incorporate two extra root stages at F and G, in order to improve on the precision of this method [17]. Therefore, to improve on the accuracy at the 16-year-cut off in this population might require the adoption of these extra stages.

The findings from this study further support the emphasis made by other authors on the need for country-specific references with respect to the use of this method for age estimation [12, 13]. From our observations summarised in Table 2, we note that the urban Ugandan population appears to mature faster than the black South Africans using the same method [10, 16]. Therefore, this Ugandan population also matures dentally faster than the Caucasians and Mongolians [10, 26]. These inter-population differences in dental development further justify the need for population-specific cut-off reference values to address various medico-legal scenarios.

Considering the fact that over 60% of minors in Uganda lack birth documentation [28], age estimation becomes a necessary practice in the Ugandan setting. For purposes of achieving more credible estimates especially for high stake purposes, a cost–benefit analysis would be recommended to inform the planning and policy-making for age estimation processes. This would demand that more resources are allocated to this area of expertise as well as local research so that we reap the maximum benefits of such techniques. This could be achieved if efforts are combined with different advocacy, human rights groups, and sports bodies, whose work involves age determination of subjects.

In conclusion, the results of this study suggest that Demirjian’s classification for the lower third molars is useful for age estimation for the specific legal ages in the 10–22 year age-group in the urban Ugandan population. The findings also reveal that determining the age at the 16-year cut-off might require the modified Demirjian method with additional root stages.

## Limitations

The study limitations include: the fact that that the samples obtained were from urban and peri-urban populations may not be a fair socio-economic representation of the whole Ugandan population. However, the urban dwellings in Kampala are composed of a predominantly transitional population in terms of rural to urban migration from different parts of the country. Thus, while the study population differs from the rural population economically, it is fairly representative of the Ugandan population with respect to the genetic mix represented by different tribal groups. Also, the use of secondary data means that we were unable to control for systemic illnesses and nutrition. Though more recent research studies demonstrate that malnutrition might have no effect on mineralisation [6]. Overall, based on the observed positive LRs being greater than one [29], implies that with this method, an individual identified as of a specified age is most likely that age or older.

## Supplementary information

**Additional file 1.** Reference data of Ugandan young people_de-identified. This data file presents age, sex and Demirjian’s classification of the lower third molar among Ugandan young people. The data was used to obtain reference values of dental age.

**Additional file 2.** Test data of Ugandan young people_de-identified. This data file presents age, sex and Demirjian’s classification of the lower third molar among Ugandan young people. This data was used to test the accuracy of the reference values of dental age obtained using Demirjian’s classification of the lower third molar.

## Data Availability

The data sets supporting the conclusions of this article are included within the article and its additional files.

## Abbreviations

AUC: area under the curve
CI: confidence interval
F-Pos: false positive
F-Neg: false negative
IQR: inter-quartile range
LQ: lower quartile
PAN: panoramic radiograph
ROC: receiver operator curve
SD: standard deviation
UQ: upper quartile
